# “A balancing act”: parents’ longitudinal perspectives of weight-related discussions with their children following obesity treatment

**DOI:** 10.1186/s12889-024-19195-1

**Published:** 2024-06-25

**Authors:** My Sjunnestrand, Nicklas Neuman, Kajsa Järvholm, Anna Ek, Karin Nordin, Ximena Ramos Salas, Karin Eli, Paulina Nowicka

**Affiliations:** 1 Department of Food Studies, Nutrition and Dietetics, Uppsala University, Uppsala, Sweden; 2 Department of Psychology, Lund University, Lund, Sweden; 3 Department of Clinical Science, Intervention and Technology (CLINTEC), Karolinska Institutet, Stockholm, Sweden; 4 European Association for the Study of Obesity, United Kingdom and Bias 180, Ottawa, Canada; 5 Warwick Medical School, University of Warwick, Coventry, UK

**Keywords:** Childhood obesity, Weight-related conversations, Qualitative research, Thematic analysis, Obesity stigma, Weight bias

## Abstract

Weight-related discussions during childhood may have long-lasting effects on children’s body image and well-being. However, little is known about how parents frame these discussions with children who have undergone treatment for obesity. Our study aimed to explore how parents perceive weight-related discussions, several years after their children started obesity treatment. This qualitative study is part of the 4-year follow-up of the More and Less study, a randomized controlled trial examining the effectiveness of a parental support program as part of obesity treatment for preschool-aged children in Stockholm, Sweden. Semi-structured interviews were conducted with 33 parents (79% mothers, 48% with a university degree, 47% with foreign background) of 33 children (mean age 9.3 years (SD 0.7), 46% girls), transcribed and analyzed using realist informed thematic analysis. Three main themes, encompassing three subthemes were developed. Under the first theme, *Parental attitudes and concerns*, parents emphasized the importance of discussing weight and health behaviors with their children, yet found it challenging due to uncertainties about how to approach it safely and sensitively. A few parents found the conversation manageable, citing their own experiences of having overweight or their style of communication with the child as facilitating the conversation. Under the second theme, *The significance of time and context*, parents said they engaged in weight-related conversations with their children more frequently as the children matured, driven by their growing self-awareness. Parents also expressed how contextual factors, such as gender and the presence of others, shaped conversations. Parents perceived boys as more resilient, thus exposing them to more negative weight talk. The third theme, *Navigating weight stigma*, revealed how parents employed strategies such as nurturing their children’s self-confidence, downplaying the significance of appearance and emphasizing health when discussing weight to shield their children from weight stigma. Taken together, we found that many parents need support to navigate weight-related discussions. Addressing weight stigma is part of children’s obesity management process, as children may be bullied, teased, or experience discrimination in different social settings. More research is needed to explore how young children undergoing obesity treatment experience weight stigma and to understand gendered differences in these experiences.

**Supplementary Information:**

The online version contains supplementary material available at 10.1186/s12889-024-19195-1.

## Background

Weight-related discussions may profoundly affect children’s body image and well-being [1, 2], but little is known about how parents frame these discussions with children who have undergone obesity treatment of any type. As early detection and treatment of obesity is crucial to prevent future co-morbidities, recent recommendations from the American Academy of Pediatrics (AAP) advocate for the initiation of treatment as early as 2 years of age [3]. At this age, treatment primarily focuses on lifestyle support, revolves around parental involvement, and often requires minimal participation from the child. Consequently, parents are required to translate the treatment into everyday actions in their home, potentially prompting discussions about weight with their children.

However, due to the sensitive nature of childhood obesity, parents of children with overweight or obesity may be reluctant to engage in childhood obesity treatment and to handle discussions about their children’s body weight. Despite recent evidence showing no association between professional obesity treatment and risk of eating disorders [4], many parents are concerned that addressing obesity might harm their children’s well-being [5, 6], contribute to unhealthy weight fixation and trigger eating disorders [1, 7]. This reluctance may explain why parents and grandparents often use euphemistic expressions when referring to their child’s or grandchild’s excess weight, such as ‘cute’ or ‘healthy’ [8]. Importantly, weight-related conversations can take various forms, and previous research has shown that the outcomes of such conversations are determined by the approach parents take [1].

Encouraging weight loss or commenting on eating habits has been associated with unhealthy weight control behaviors, disordered eating [9, 10] and excess weight [11] in adolescents, which may last into adulthood [12]. Mothers’ negative comments about their own or others’ weight or body size negatively impact adolescents’ self-worth [13] and body image [14]. Moreover, parents’ weight-related discussions with their children may involve weight-based teasing, with severe implications for their mental health and overall well-being [2]. This includes an increased risk of developing mental health problems, including depressive symptoms [15, 16], psychological distress, anxiety [17] and suicidal thoughts and attempts [18].

On the other hand, weight-neutral discussions that focus on health behaviors rather than body weight may have positive effects on children’s well-being [1, 19]. Such approaches may also protect against disordered eating and dieting in adolescents, as compared to avoiding the subject or solely focusing on body weight [20]. Similarly, conversations in which parents emphasize healthy eating rather than weight are linked to a reduced risk of unhealthy weight-control behaviors and dieting among adolescents with overweight [10]. However, as previous studies on weight-related conversations within the home have mostly focused on adolescents, little is known about how parents frame such conversations with younger children. It is crucial to uncover this, as early childhood is a critical period for the development of body image and younger children may be particularly vulnerable to how those around them, including their parents, speak about body weight and size [21]. Understanding how parents perceive weight-related conversations with children as they grow from preschoolers to pre-adolescents is essential to foster positive conversations around body weight.

This study addresses a present gap in the literature by exploring how parents experience and shape weight-related discussions with their children, several years after the children started obesity treatment. It explores how parents of children who underwent obesity treatment approach and experience weight-related discussions, and thus provides a unique retrospective account of parents’ evolving experiences of weight-related discussions, from the time of the obesity intervention, when their children were 4–6 years old, to the time of the interview, 4 years later. In our previous publication [22], based on interviews conducted immediately after the obesity treatment, parents expressed social and emotional challenges related to handling their child’s obesity, managing their child’s appetite, seeking obesity treatment, and coping with the social stigma surrounding obesity. Here, we further explore these, and other aspects of parents’ experiences, in depth.

## Methods

### The More and Less study

The data for this study were collected as part of a 4-year follow-up of the More and Less (ML) study, a randomized controlled trial (RCT) examining the effectiveness of a parent support program as treatment of obesity in preschool-aged children in Stockholm, Sweden [23]. Between 2012 and 2016, parents of 177 children identified as having obesity according to age and sex-adjusted reference data [24] participated. The participants were randomly assigned to one of three groups: a 10-week parental support program (1.5 h/week); the same program with additional follow-up sessions (boosters); or standard treatment offered at an outpatient pediatric clinic. Half of the participants received the parental support intervention, covering supportive parenting practices and lifestyle behaviors. These sessions required participation from parents only, hence, no children were present. Those who had been allocated to additional follow-up sessions received a booster telephone call every 4–6 weeks of the remaining 9 months.

Participants in the standard treatment group received at least 4 visits at a pediatric outpatient clinic focusing on food and physical activity advice, with the child present. A more detailed description of the study has been published elsewhere [23]. Four years after study start, all parents were invited to take part in follow-up interviews to explore long-term experiences of managing their children’s weight. These interviews constitute the data for this study. The interviews are treated as a single set, without comparison between groups, as the focus is on parents’ experiences rather than the RCT.

### Recruitment and participants

Families from the first two years of the ML program (*n* = 67) were contacted by telephone and invited to participate in the interviews. Fourteen families (21%) could not be reached, 2 families (3%) had moved abroad, and 18 families (27%) declined participation. Reasons for declining participation included lack of interest, family reasons (illness, separation), a preference not to discuss their child’s weight, language barriers, and a lack of time. Hence, a total of 33 families agreed to participate in the interviews. All participants received a participant information sheet and signed an informed consent form. Table 1 outlines the characteristics of the participating families at the time of interview. The anthropometric measures were conducted at a clinic and obtained from data collected during the More and Less study.

**Table 1:** Characteristics of the included families in this interview study

	*N* = 33
	*N* (%)
Parent support group	16 (49)
Standard treatment	17 (52)
*Children*	Mean value (SD)
Age (years)	9.3 (0.7)
Weight category	*N* (%)
Normal weight	1 (3)
Overweight	12 (36)
Obesity	10 (30)
Severe obesity	10 (39)
Sex	
Female	15 (46)
Male	18 (54)
*Parents*	Mean age (SD)
Age (years)	41.1 (5.6)
BMI (kg/m^2^)	28.6 (5.6)
Sex	*N* (%)
Female	26 (79)
Male	7 (21)
Foreign background	
Yes	14 (47)
No	16 (53)
Weight category	
Normal weight	6 (18)
Overweight	12 (36)
Obesity	8 (24)
Missing	7 (21)
Highest school grade	
Grade school	1 (3)
High school	13 (39)
College/University	16 (48)
Missing	3 (9)

Abbreviations: SD, standard deviation; BMI, Body mass index

Foreign background: parent and both grandparents born in a country other than Sweden or parent born in Sweden and grandparents born abroad

Parents were classified as having normal weight, overweight or obesity according to the World Health Organization’s cut-off criteria for BMI (WHO, 2019)

### Data collection

Individual, semi-structured interviews were conducted by KN, a female pediatric nurse and research assistant with extensive experience in qualitive research. The interviews followed an interview guide developed by an expert team consisting of KN, four senior researchers with expertise in parenting and childhood obesity, and one doctoral student. The expert team created an initial draft of the interview guide where each question was evaluated using Content Validity Index (CVI). CVI is an assessment tool that can be used to measure the question’s relevance in relation to the concept the questionnaire is intended to study [32]. In order to evaluate CVI for the questions in the interview guide, each member of the expert group filled out a form to assess the relevance of the selected questions (See Supplementary file 1). All expert opinions were collected, and based on these, the CVI was calculated for each item (I-CVI), as well as for each subscale (S-CVI, mean), following the recommendations of Polit & Beck (2006).

The interview guide focused on lifestyle changes following participation in the ML program (i.e. “What resources that your family has been offered related to your child’s weight have been helpful?”, “What lifestyle changes related to diet and physical activity has your family made during the last four years?”, “Who has been involved in your child’s obesity management?”). Questions were constructed to capture parental experiences of having a child aged 8–10 years who might still have overweight or obesity (i.e. “In what way has your child’s body image changed over the past four years?”, “Has it become easier or more difficult to deal with these questions, in what way?”, “How do you talk about your child’s weight with him/her?”, “What do you think is important to consider in such conversations?”, “How would you say it?”). Follow-up questions were asked based on the parents’ responses and field notes were made during the interview. The interview guide was pilot tested on one of the participants, however no changes were deemed necessary. For the participants’ convenience, all interviews took place over the telephone. Only KN and the participant were present during the interviews, which lasted 28–70 min (45 min on average). The interviews were audio recorded and transcribed verbatim by two undergraduate students. The reporting of this study follows the Consolidated criteria for REporting Qualitative research (COREQ) checklist (See Supplementary file 2).

### Data analysis

The interviews were analyzed using realist informed thematic analysis according to Braun and Clarke [33]. Thematic analysis is a qualitative research method for identifying, analyzing, and reporting patterns or themes within data. All the transcribed interviews were read by two authors (MS and PN). As the interviews included parental perspectives on both the obesity intervention and parenting a child with overweight/obesity, we first extracted only the interview data concerning parenting. MS and PN screened the first five interviews and MS screened the rest of the interviews. This enabled us to focus our inductive analysis only on data relevant to the research question. When this first screening was finished, the material was re-read, extracted into an Excel spreadsheet and coded by MS using an inductive approach. An inductive approach allows the researcher to develop data-driven codes and themes, rather than work with a pre-determined coding frame [33]. When half of the interviews were coded, MS, PN, and NN met and discussed potential themes based on the codes. Following this, MS coded the remaining material and regularly met with PN and NN to discuss and refine the themes. The proposed themes were documented and shared twice with all co-authors. The wider team’s critical feedback was used to revise the themes until consensus was reached by all co-authors.

## Results

### Themes

**Fig. 1:**
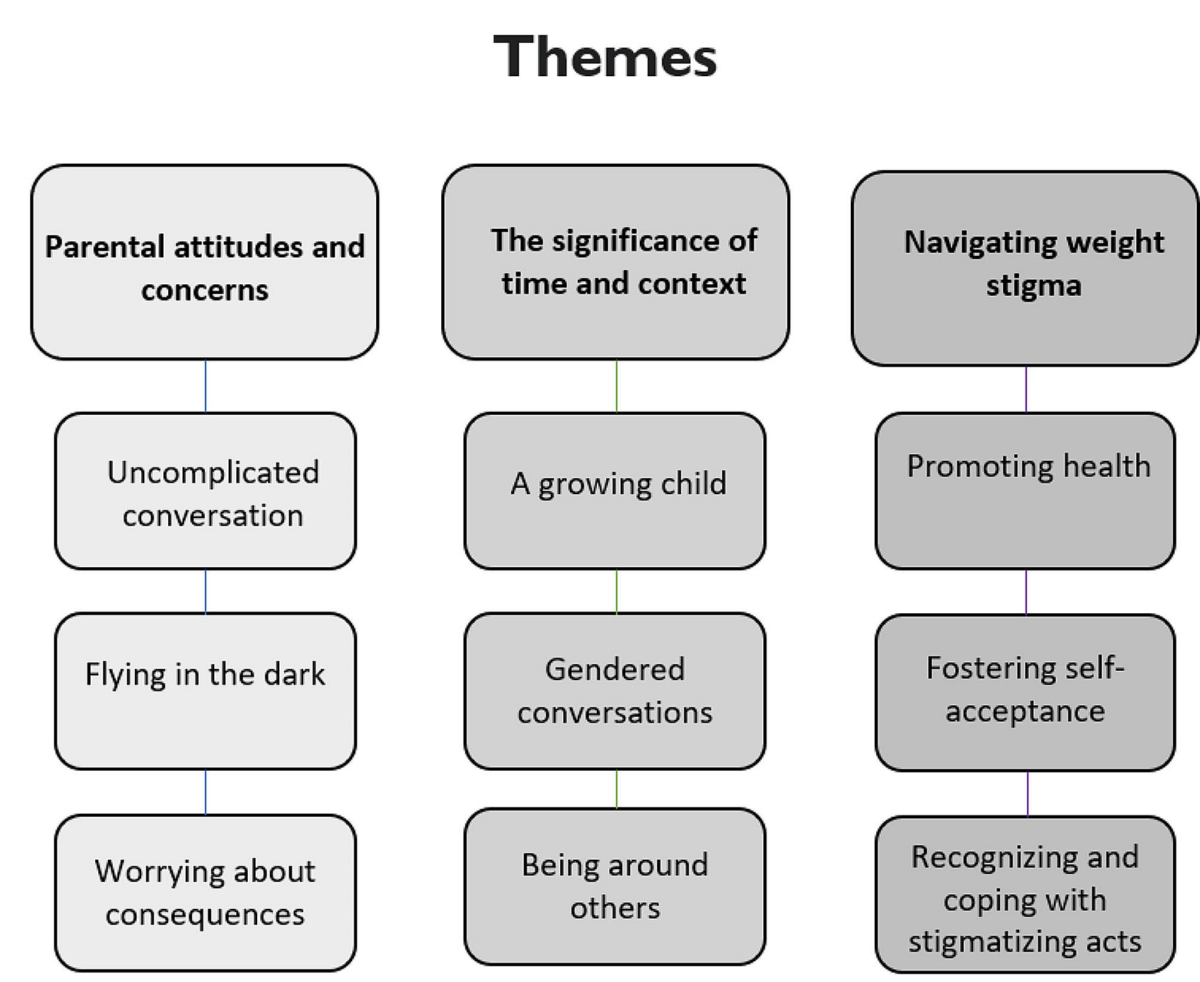
Identified themes from the thematic analysis

Three main themes, encompassing three subthemes each, were developed. These are presented in Fig. 1 and described in detail below. Each theme includes illustrative quotes from parents (M for mother; F for father), followed by a personal identification code.

### Parental attitudes and concerns

This theme revolves around parents’ emotions and uncertainties regarding weight-related conversations. The theme encompasses three subthemes: *Uncomplicated conversation, Flying in the dark*, and *Worrying about consequences.*

### Uncomplicated conversation

Several parents emphasized the importance of having weight-related discussions with their children, recognizing this as a way to help them develop necessary and sustainable self-care skills to manage their obesity. However, their approaches differed markedly. One mother put it as: *“You teach the child to walk and you teach the child to eat with cutlery, you also have to teach the children how to manage their sensitivity to excess calories”* (code M23). Thus, helping the child understand her “calorie sensitivity” was compared to any other life skill, adding to the list of responsibilities for parents.

Furthermore, as many of the children had dealt with overweight for many years, parents found themselves having such conversations with their children several times. *“I have probably learned along the way how to handle it”*, one mother said (code M12). *“Throughout the journey”*, as she put it, she gradually became more at ease with having weight-related discussions with her son. This mother, and a few other parents sharing similar views, tended to perceive the conversation as less challenging. The parents, particularly those who had personal experiences of overweight, expressed that having an open and trusting relationship with their child helped these conversations. One mother, for instance, considered this conversation part of a general openness in her relationship with her child:

*No, I don’t think it’s challenging [to talk to my son about his weight]. I have been in that situation myself. We are open with each other, and we have always had that relationship. (code M27)*

Several parents also said that speaking with other parents about how to approach weight-related conversations helped them gain confidence, suggesting that sensitive conversations could be aided by social support.

Experiencing weight-related conversations as uncomplicated did not mean that parents used weight-neutral approaches. A few parents directly commented on their child’s appearance, telling them that they were overweight and emphasizing the need for change, by pointing out that the child’s clothes no longer fit, or by showing them pictures where they appeared thinner. They believed that making their child aware of their excess weight was a good step in motivating them to change their health behaviors. Moreover, some parents conveyed that having overweight was undesirable and should be avoided, indicating the presence of weight bias, as described by this father:

*He once said very angrily and annoyed that he doesn’t care about what he looks like and what people talk about, this was after his mother had explained that it wasn’t good for him to look like that. ‘Look at you’ [she said] […] He can’t wear clothes, normal clothes, because the waist is huge and we buy trousers in three sizes bigger, they are too long and he knows that it’s a problem […] He will probably accept it eventually, understand that being overweight, being strong and big is one thing, but just being fat and stuff like that… He should have muscles, as big muscles as he can get, but not the belly, big belly, waist, fat… yes. He… sometimes, it feels like he is accepting it [eating less], but when he sees food, he seems to forget everything. (code F33)*

### Flying in the dark

Several parents experienced a great deal of uncertainty and worry about weight-related conversations, saying they found these sensitive and difficult, with some feeling reluctant to speak about weight at all. As one mother succinctly put it: *“It’s always a balancing act. How much you should talk about it.”* (code M87).

Parents experienced a dilemma between wanting to help their children with weight management and fearing they might harm them in doing so. This concern, as well as an uncertainty about when and how often to raise the discussion and what language to use, was often cited as a barrier to the conversation.

*It’s just that it’s so sensitive and I don’t want it to have consequences and I don’t want her to feel like there’s something wrong with her. She’s totally aware that she’s bigger than everyone else, I don’t want to tell her things that are obvious. (code M67)*

Some parents expressed concerns about being mean to the child, feeling that the discussions created a negative atmosphere and led to constant nagging. Additionally, some parents reported feeling frustrated during weight-related conversations, often arising from the uncertainty of how to navigate the sensitive topic. This frustration occasionally led parents losing their patience and saying things they later regretted.

*You often get frustrated and then you might say things you don’t mean. Because you don’t know how to say it sometimes. He doesn’t really understand either and sometimes you have to speak clearly. And then you feel mean. (code M87)*

### Worrying about consequences

Several parents expressed worries over weight-related conversations negatively affecting their children. Specifically, parents worried their children would become preoccupied with food intake, weight, and appearance. *“I don’t want him to be like seven or eight years and already thinking about getting chubby by what he puts in his mouth”*, a mother said (code M12). This quote is representative of several parents who referred to their children’s young age as a crucial period for body image development, expressing concerns about their children being concerned about their weight so early in life. Parents feared that excessive emphasis on their child’s weight and eating habits could potentially trigger unhealthy thoughts and contribute to a distorted body image. A mother said:

*He can be like that sometimes, asking questions like ‘Is this unhealthy, is this healthy?‘, you know, to the point where I can almost become a bit worried sometimes, that I’m nagging too much. I just want what’s best for him, but at the same time, I don’t want it to go to the extent where he starts feeling bad about his eating habits and his appearance. (code M87)*

Worries about children developing an unhealthy body image or obsession over food ultimately boiled down to parents’ fear of triggering eating disorders, especially among girls. In relation to this, a few parents also shared personal experiences of having had eating disorders in their youth due to weight-related comments from others.

Lastly, a few parents said that their uncertainty about addressing weight-related issues with their children in a safe and sensitive manner led them to avoid the conversation completely. Instead, they attempted to manage their child’s weight without their awareness or involvement. *“I’ve had some issues myself, and I was also overweight as a child. So, I don’t want to discuss it too much because I know it can turn in the other direction and become just as difficult or even more so”*, one mother said (code M25). Thus, her personal negative experiences with frequent weight-related conversations in her youth made her cautious about discussing it extensively with her own son, concerned about possible adverse consequences.

### The significance of time and context

This theme includes parental perspectives on how they perceived and adapted weight-related discussions with their children in response to contextual factors. Within this theme, three subthemes were identified: *A growing child, Gendered conversations*, and *Being around others.*

### A growing child

As four years had elapsed since the families started obesity treatment, parents reflected on how the child’s becoming older affected weight conversations. Several parents said they had noted a gradual increase in their children’s self-awareness, a trait less prevalent during their younger years. A mother said: *“On a few occasions, for instance, he stood in front of the mirror […] and then he looked at himself, and he said, ‘Oh, I’m big and fat,’ you know, like that.”* (code M87). Similar experiences were shared by several parents, noticing how their children had started to compare themselves to peers and express self-criticism or insecurity about their appearance.

Consequently, parents observed how their children began attaching greater importance to their clothing style and overall appearance, sometimes feeling uncomfortable in situations like sports, showering, or changing clothes in front of others. Some even employed strategies to appear slimmer, as illustrated by this mother’s account:

*What I can see is that sometimes, in front of the mirror when she thinks I’m not watching, she tries on a shirt that’s too small and sucks in her stomach, trying to look thinner than she is. […] I notice how she’s figured out ways to look slimmer, so it’s clear that she’s fully aware that she’s bigger than all the other children (code M67)*.

As children became increasingly self-aware about their weight and appearance, parents noted that they engaged in weight-related conversations with them more frequently than before. Some said they had recently started to have these conversations. Given that many children had become more self-conscious about their appearance, according to the parents, some felt that the discussions had become more challenging over the years.

*As he grows older, it gets more challenging, because he’s also more aware. Both in terms of… I mean, body perception and how it affects him emotionally, not just, what should I say, the purely practical or theoretical aspect” ( code M72)*.

In contrast, some parents found that as their child grew older and matured, the conversation became easier. Now they felt that they could engage their children differently as they were more cognitively developed. Other parents acknowledged the mixed aspects of their child’s age, as expressed by this mother:

*Yes, it’s easier to talk to them when they’re older because they understand a lot more. However, I find that they’re a lot less easily influenced when they’re older because they have their own thoughts, and I know from my own experience how I used to think like, “Mom is being silly” [laughs]. So, in that sense, it’s quite difficult to influence them (code M5).*

### Gendered conversations

In addition to the temporal aspect, parents reflected on how the nature and dynamic of weight-related conversations was influenced by their child’s gender. Both mothers and fathers acknowledged being more cautious when discussing weight-related issues with their daughters compared to their sons, based on a general perception that girls are more vulnerable to such conversations. According to some parents, this could be attributed to the greater societal pressure on girls regarding thinness and appearance. A few parents also expressed the belief that girls are inherently more sensitive than boys, while asserting that boys should be able to handle weight-related discussions with less concern. *“[Girls] are more sensitive … they take it more seriously”*, one mother said (code M7).

Another mother shared this view and expressed relief over being mother to a boy, implying that she had fewer concerns about her son’s body image compared to if she had been raising daughters:

*Now, I’m lucky since it’s a boy. I imagine it can be even worse for girls … Then, of course, when it comes to comparing themselves against others… I get the feeling that boys don’t do it in the same way. (code M72)*

Of note, throughout the interviews, most parents who discussed body image at length did so in reference to their sons; however, they expressed explicit concerns about body image issues and disordered eating mostly in relation to girls.

### Being around others

Parents found it challenging to attend to and limit their child’s food intake in social situations such as parties or gatherings.

*It doesn’t feel good when you’re away, having to say in front of everyone ‘No, now you can’t eat any more’ and stuff like that. It can be a little … I feel it can be a little sensitive for her. (code M14)*

Several parents found it difficult to limit their child’s food intake in front of other children. They expressed discomfort in instructing their child to avoid excessive intake of food and sweets in these situations, as they feared it would draw attention to their child and cause feelings of shame. This was perceived as particularly challenging when being around children without food intake restrictions. A few parents noted that their children compared their food intake to others, showing awareness that other children were allowed to consume larger quantities of food. A mother said: “*She can reflect on [her] friends sometimes eating much more than her, saying ‘These friends can eat so much candy, mom, it’s really strange!‘*” (code M58). Such situations could also prompt questions from the child:

*Like, the cousins get to have more, they’re really thin, they have trouble gaining weight, it’s the opposite there. [Her son says] ‘Why does he get to eat…?’ What should I say, ‘He hasn’t eaten all day’? […] It becomes difficult in such situations (code M27)*.

This last quote illustrated the mother’s balancing act of selecting the right words to use. Her son felt confused in the company of other children without dietary restrictions, and the mother found herself searching for the appropriate response.

### Navigating weight stigma

This theme explores how parents navigated the social stigma associated with obesity, highlighting positive framings parents used to counteract stigma and boost their children’s wellbeing and self-esteem. It encompasses three subthemes: *Promoting health, Fostering self-acceptance*, and *Recognizing and coping with stigmatizing acts.*

### Promoting health

For several parents, weight-related conversations with their children primarily focused on overall health. Parents addressed potential long-term health implications of excessive weight gain or highlighted the importance of dental health and physiological well-being. Some parents also educated their children about healthy and unhealthy food choices and how these choices impact the body, for example, saying that food provides the body with energy, but that the body needs *“the right [type of] energy”* (code M58). Moreover, they emphasized the physical aspects of weight, explaining that excess weight can make it more difficult to engage in activities such as playing, running, jumping, and climbing. Like a mother who stated that *“[w]e’re discussing it in terms of your ability to keep up with school, to participate in sports, and to feel well” (code M58).*

To reduce feelings of blame and stigmatization, parents referred to the well-being of the whole family. “*We talk a lot about what we as a family need to feel good and be healthy*”, another mother concluded, “*to approach this from a larger perspective*” (code M21). This view was shared by several parents, suggesting that they actively tried not to make their child the focus of weight-related conversations.

Moreover, some parents emphasized the importance of approaching their children in a sensitive and non-judgmental way and avoided negative, possibly stigmatizing language. They did so by refraining from mentioning appearance or body size. A mother shared that emphasizing the child’s health rather than appearance or size made weight-related conversations easier:

*I believe that it’s easier as a parent to talk about health from within and not, I mean, that’s what one should strive for. Instead of how it looks on the scale or how it looks in clothes and waist measurements. (code M12)*

### Fostering self-acceptance

Parents who engaged in weight-related discussions that focused on health, rather than size or appearance, often said they wanted their children to accept themselves as they were regardless of weight status. Several parents emphasized the importance of fostering confidence and self-acceptance by alluding to diversity, such as this mother:

*He’s well aware that he’s a bit bigger than the others and… Yes, for a while he wanted to wear a t-shirt in the public swimming pool. Because he thought he had like big boobs. Eh, but then I kept telling him that ‘We all look different and there are thin ones, there are chubby ones, there are dark-skinned ones…’ and I sort of tried to give him that overall picture that everyone doesn’t look the same. And he has sort of accepted that. (code M7)*

This mother tried to convey the message that people look different and that this is fine. Several parents further encouraged their children to focus on what the body can do rather than fixating on their looks, thereby downplaying the significance of weight and appearance as the primary indicators of a person’s value or self-esteem. Additionally, some parents tried to communicate that being thin does not equal happiness or good health.

Parents tried to promote this self-acceptance through acting as role models and fostering a positive and non-judgmental familial environment. For example, a few parents mentioned avoiding discussions and stigmatizing comments related to people’s body sizes or appearances. Others tried to boost their children’s self-confidence by setting an example for their children, consciously avoiding making negative comments about their own weight and appearance but rather complementing themselves. A mother shared an example of how she and the child’s father had boosted their son’s self-confidence by making positive comments about themselves in front of the child.

*Both me and [the son’s father] are role models …. [My son] can look in the mirror and say ‘God, I’m handsome’ because [the father] does that too [laughs]. Maybe I don’t do it as often, but I still try to remember to avoid talking negatively about my body in front of the kids. I try to avoid that when I talk to myself too. To stand in front of the mirror and think negatively about yourself or that some part of your body looks bad. Definitely not in front of the children, I want them to feel good about themselves regardless of how they look. (code M70)*

### Recognizing and coping with stigmatizing acts

Almost all parents recalled situations when their children had encountered weight-based *stigmatizing acts*, that is “specific negative behaviors or communications involved in stigmatizing actions or events” [34]. One example was weight-based teasing or bullying from school peers. Such incidents often left their children feeling distressed. In response, parents sought to comfort their children, emphasizing that those engaging in such behavior might have their own insecurities, and encouraged their children to consult the school staff. Some parents took proactive steps by scheduling meetings with their children’s school teachers to address the issue.

According to several parents, weight stigma extended beyond the school setting. Some parents recounted situations where other family members, such as siblings and grandparents, made well-intended but thoughtless comments about the child’s weight. For example, one mother recalled her daughter’s older brother noticing his little sister’s weight when they had to be weighed when renting skis during a trip, commenting: “*’Oh, you really weigh a lot!*’” (code M22). A common strategy parents employed to cope with these situations was to talk to their family members, conveying that such comments were not appropriate or acceptable.

A few parents also described distressing encounters in clinical settings, where healthcare professionals expressed negative attitudes towards the parent and the child due to the child’s weight, effectively stigmatizing both the “bad” parenting and the weight itself. These incidents sometimes occurred in the presence of the child: “*We encountered a terrible doctor*,” a mother told the interviewer, “*who said to her something like, ‘If you continue like this, you’ll weigh 130 kilos by the time you’re a teenager, and you wouldn’t want that*’” (code M88). Such incidents could lead the parents to cancel follow-up healthcare appointments in an attempt to protect their children from stigmatizing acts. According to the parents, these experiences increased their children’s distress, with children becoming self-conscious about their appearance and not wanting to eat too much to avoid gaining weight.

## Discussion

Our study is the first to explore how parents discuss weight-related issues with their children, several years after participation in a childhood obesity treatment program. While parents emphasized the importance of discussing weight and health related behaviors with their children, they found it challenging due to uncertainties about how to approach it safely and sensitively. A few parents found the conversation manageable, citing their own experiences of having overweight or their style of communication with their child as facilitating the conversation. Some parents said they engaged in weight-related conversations with their children more frequently as they matured, driven by children’s growing self-awareness. Parents also said that contextual factors, such as gender and the presence of others, shaped the conversation. In particular, parents perceived boys as more resilient, thus exposing them to more negative weight talk. Lastly, parents employed strategies such as fostering their children’s self-confidence, downplaying the significance of appearance and emphasizing health when discussing weight with their children to shield them from weight stigma. Below, we discuss three overarching aspects of these findings, and their implications.

Our findings highlight that parents balance between managing their children’s obesity on the one hand, and the need to protect their well-being on the other. This aligns with previous qualitative research showing that parents find themselves caught in a moral dilemma between these two objectives [35, 36] and that communication about weight and weight-related behaviors is perceived as sensitive and delicate [27–38]. Consequently, parents may find themselves lacking the necessary knowledge and support required to navigate these conversations effectively. Similar findings were raised in our previous qualitative study, in which parents expressed a need for increased guidance and support from healthcare professionals [5]. This absence of support to address their child’s weight may foster a sense of hopelessness, as shown in studies with parents of older children with obesity [39, 40]. That is, parents may develop the belief that children will struggle with obesity for the rest of their lives. While this finding was not present in our study, it is important to note that the children in our study are still young. Thus, providing adequate support to parents, strengthening their self-confidence and instilling hope is crucial.

Despite parents expressing uncertainties about how to navigate weight-related conversations effectively, this study provides several good examples of such discussions. For instance, some parents mentioned employing a weight-neutral approach and redirected the conversation away from size and appearance. This type of approach has been linked to improved well-being [1, 19] and a decreased risk of eating disorders in adolescents [11].

Other accounts shed light on a more problematic approach in weight-related discussions with children and reveal noticeably weight-biased attitudes among a few parents. Specifically, some parents described employing negative and judgmental language as a means of motivating their children to lose weight. This aligns with previous research that highlighted parents as a potential source of weight stigma [2, 41]. Indeed, an earlier study found that parents may speak about children with obesity as lazy and lacking self-control [41], implying a belief that their children’s excess weight stems from personal flaws. The fundamental starting point in coping with stigmatizing acts, according to Sobal, is recognition [34]. Once obesity is recognized as stigmatized, it can be countered. Given that parents are key resources in obesity treatment, providing guidance and support for safe and sensitive weight-related communication is a vital priority. However, there is a lack of evidence-based guidelines for how such communication is best carried out.

In all forms of childhood obesity treatment, parents need support to understand that while obesity is a chronic disease [26] that may require lifelong treatment, children with obesity can live healthy lives and develop healthy relationships with their bodies. Living with a chronic disease like obesity does not mean that children will suffer for the rest of their lives. If children receive obesity treatment which emphasizes improvements in health and quality of life, they can develop the lifelong skills necessary to live well with obesity [42]. In our study, while some parents understood these messages, others did not. The parent support program offered in the ML study emphasized the children with obesity can lead healthy lives. However, the participants’ mixed endorsement of this message conveys the importance of reinforcing it throughout obesity treatment and beyond. To achieve this, it may be helpful to train pediatric healthcare professionals with whom families engage outside the specialized obesity treatment context.

In a few cases, parents spoke of their children encountering stigmatizing acts from healthcare professionals. This aligns with research showing that weight bias attitudes are prevalent among healthcare professionals, including pediatricians [28, 43]. This is concerning, as parents conveyed that such instances prompted them to avoid future obesity management appointments, posing a potential risk of delaying or even preventing appropriate treatment – a risk that has been observed in previous research [28, 44]. For example, a study by Puhl et al. revealed that almost a quarter of parents who perceived their doctor using stigmatizing language in relation to their child’s weight reported they would cancel any future healthcare appointments [45]. In addition, primary healthcare professionals’ minimization of parents’ concerns poses a barrier to childhood obesity treatment [39]. Given the recognized effectiveness of early treatment [46], providing training and tools for healthcare professionals to communicate with parents in a compassionate, sensitive, and non-judgmental way is of paramount importance.

Lastly, most parents expressed that girls were more vulnerable to weight-related conversations than boys, and it appeared that this gendered perception affected the sensitivity with which they approached these conversations. This finding corroborates existing but limited research suggesting that parents seem to be more inclined to engage in negative weight-related conversations with their sons rather than their daughters [47]. Interestingly, however, despite an almost equal distribution of parents with sons and daughters in our sample, the accounts related to body image in this study tended to focus more on boys. This was unexpected, as parents in general expressed more concerns about girls. Crucially, there is no evidence suggesting that boys are immune to the negative effects of weight-related conversations [1]. The perception that boys are more resilient than girls may inadvertently render boys’ distress invisible and contribute to the internalization of weight stigma. This should raise some caution about a potentially gender-biased misconception of boys not being hurt by weight stigma, or perhaps even a cultural norm that “a boy should be able to take it” (hinted in some parents’ stories). It is important to understand the potential consequences of such attitudes for boys’ body image and self-esteem.

### Strengths and limitations

One of the strengths of this study is the relatively large sample size (for a qualitative study) and the diversity of participants in terms of both the parents’ and children’s characteristics. However, as with all qualitative studies, this study represents the subjective narratives of parents and may not be representative in other contexts which limits the generalizability of the findings. Furthermore, it is important to note that this study focuses on parental *accounts of* their conversations, not the conversations as such. Interview situations can influence parental responses through impression management, where individuals shape their answers to align with perceived expectations [48]. In this context, parents may believe that a specific approach to discussing their child’s weight aligns with the interviewer’s expectations, creating an uncertainty about the accuracy of their responses.

The previous interviews with parents conducted immediately after treatment mainly focused on parents’ experiences of the study [22]. If those interviews included the same set of questions used in this study, we would have been able to capture their longitudinal experiences of raising a child in the context of obesity treatment. Furthermore, how the children themselves perceived weight-related conversations remains unexplored, which we acknowledge as an additional limitation. Importantly, however, the children in the study have been interviewed about food and meals in their everyday lives. For ethical reasons, the interview questions did not raise the topic of weight, but the findings suggested the children had positive and joyful relationships to food and eating in company with others [49]. Lastly, as the interviews included parents from both treatment groups, no comparison between the two treatment arms was possible.

### Implications and future research

Future studies should consider including observations to analyze weight-related conversations between parents and their children. This may be extended to examine the role gender has in shaping these discussions. As this study suggests, boys may be subjected to negative weight-related discussions as their parents may perceive them as more resilient. Going forward, this emphasizes the need to further investigate how weight-related discussions can impact children, irrespective of gender. In addition, future studies should investigate whether parents employ different approaches when discussing weight with their sons compared to their daughters in larger samples of girls and boys.

Furthermore, this study reveals that children may be bullied, teased, or experience discrimination because of their weight in several social settings, including within their own family. These findings align with our previous interview study, conducted immediately after treatment, in which parents articulated feelings of blame, guilt, and judgement by healthcare professionals [5]. This underscores the pervasiveness of weight stigma and the need to address this issue on a societal level. In the United States, proposals have been made to legislate against weight stigma and discrimination [29]. Although the progress of these proposals has been slow [29], states with anti-bullying laws that specifically include weight as a protected class have witnessed a decrease in weight-based bullying among youths [30]. Therefore, extending comparable legislation to a Swedish context may potentially reduce weight-based bullying.

To ensure that the healthcare settings remain free of prejudice and discrimination, countering weight bias among healthcare professionals is critical. However, current initiatives to reduce weight stigma have shown, at best, moderate effectiveness [31]. This underscores the need to develop more effective interventions targeting healthcare settings, as also emphasized in recent recommendations from the World Obesity Federation [25]. Notably, interventions directed at students in health-related professionals show more promising results in reducing weight bias [50]. Therefore, implementing comprehensive interventions early and throughout healthcare education may be beneficial.

## Conclusion

In this unique study, we conducted interviews with parents to explore their long-term experiences of navigating weight-related conversations with their growing children post-obesity treatment. We found that many parents need support to navigate weight-related discussions as they express uncertainties regarding how such communication can be done in a safe and sensitive manner. In general, parents perceived boys as more resilient, potentially placing boys at a greater risk of exposure to negative weight-related discussions. Lastly, addressing weight stigma is part of children’s obesity management process, as children may be bullied, teased, or experience discrimination in different social settings. More research is needed to explore how young children undergoing obesity treatment experience weight stigma and to understand gendered differences in these experiences. Our findings underscore the importance of developing impactful interventions to address weight stigma within the healthcare system and society at large.

### Electronic supplementary material

Below is the link to the electronic supplementary material.

Supplementary Material 1

Supplementary Material 2

## Data Availability

The transcripts/data of this qualitative study are not publicly available due to confidentiality agreements with the participants but are available from the corresponding author on reasonable request.
