# Supplementary material for: “A balancing act”: parents’ longitudinal perspectives of weight-related discussions with their children following obesity treatment
Source: BMC Public Health. 2024 Jun 25;24:1695. doi: 10.1186/s12889-024-19195-1 (PMC11202376; doi:10.1186/s12889-024-19195-1)
Supplement: Supplementary file 1 — Supplementary Material 1 [file 12889_2024_19195_MOESM1_ESM.pdf]

Assessment of Content Validity Index for questions to interview with a parent

Instructions:
Indicate with “x” how relevant each question is for reaching the overall aims with this study:
1. Understand how parents have perceived the obesity treatment of their child, both the positive and negative consequences of the treatment since the treatment ended.
2. How to be a parent to a child aged 8 to 10 years who was classified with obesity at age of 4 to 6 years and may still have obesity.

The order of the questions is not yet established. You may offer an alternative formulation of any question by adding a commentary using track changes.

	**Question**	**Not relevant**	**Less relevant**	**Quite relevant**	**Highly relevant**
1	How have you experienced the time after the initial year of the program/study?				
2	What lifestyle changes, both when it comes to physical activity and food, have you and the family done during the treatment?<br>Did you manage to maintain these changes?<br>What was the easiest?<br>What was most difficult?				
4	Looking back, which elements of the programme/treatment have been most helpful?<br>Which do you remember using at the start, and which have you retained over time?				
5	Thinking back to the time in your life just before you took part in the programme/starting treatment, do you remember how you felt and what you thought about child feeding and weight?<br>Do you feel like your knowledge and attitudes have changed since then?<br>If so, in what ways?				
7	How do you work together in the family around healthy habits?				
8	In what way have you involved the staff at school regarding your child’s weight related work?				
10	How has the program/treatment affected you and your family? Probe: Positive consequences, negative consequences.				
11	Have you or your family experienced any important changes in the four years since the programme/treatment started? (Changes may include house moves, the birth of a child, job changes, or relationships ending or starting.)				

	Do you feel like any of these changes made healthy feeding easier, or more difficult?				
12	Have issues relating to your child's body image and feeding become easier or more difficult over the last four years?				
13	How do you talk about your child's weight with him/her?<br>What do you think is important to discuss with your child regarding this topic?<br>What is important to consider when talking about overweight with children?				
14	In what way did the treatment affect your way of being a parent?<br>In what way did the program/treatment affect your feeling of being a competent parent?				
15	In what way could the treatment be altered to suit your family better regarding office visits, layout of the program/treatment?				
16	Are there any risks with the program, any negative consequences for parents or children that we should think about?				
17	Was there anything that was missing in the treatment or that it could be more of?				
18	Do you think that the treatment was worth your time and effort?				
19	What are the biggest challenges you've experienced in practicing healthy feeding?				
20	Do you have anything to add?				

Additional questions for parents who participated in the ML parent groups

You may offer an alternative formulation of any question by adding a commentary using track changes. The order of the questions is not yet established.

	**Question**	**Not relevant**	**Less relevant**	**Quite relevant**	**Highly relevant**
1	Now there have been four years since you participated in the parent groups. I wonder if there are some moments during the program that you remember especially well?<br>What was good with the parent groups?				
2	How have you used the handouts/manual/material you got during the parent group?<br>Is the material still useful even though your child is older?				
3	During the program we talked about different parental techniques, including encouragement, cooperation, schedules, setting limits, managing emotions, managing power struggles, and planning.<br><br>In what way did you find these techniques useful? Were there any techniques that were not useful? In what way?				
4	What parental techniques do you still use?				
5	What do you think is important to think about when setting limits to children?				

Assessment of Content Validity Index for questions to interview with a child

Instructions:
Indicate with “x” how relevant each question is for reaching the overall aims with this study:
To understand how a child thinks about food and physical activity/movement and how is it is to live with obesity.

Keep in mind that the questions are addressed to **children aged 8 to 10 years**. The children whose parents participated in the ML parent groups did not participate themselves in the groups. The order of the questions is not yet established. You may offer an alternative formulation of any question by adding a commentary using track changes.

	**Question**	**Not relevant**	**Less relevant**	**Quite relevant**	**Highly relevant**
1.	What do you like to eat?				
2.	What kind of food do you like?				
3.	How often do you eat your favorite food? Would you like to have your favorite food more often?				
4.	What would happen if you only had your favorite food?				
5.	What happens if something that you don’t like is served for dinner?				
6.	Sometimes parents want to teach kids new things, as for example try new veggies, how would you like them to do to make you try new things?				
7.	How does it feel to be hungry, do you think?				
8.	How often are you hungry?<br>Or how often do you think about food?				
9.	Do you think more about food when you are happy, sad, tired?				
10.	What happens if you are hungry but you can’t have anything to eat because dinner will be served in a little while?				
11.	If you were to describe to be how it feels to be full, what would you say then?				
12	Do you often feel full?				
13.	What does your parents say if you ask for an extra portion for dinner?				

14.	Is there something you are not allowed to eat? Never? As often as you would like?				
15.	If you compare to your friends, do you eat more or less than your friends?				
16.	Who do you eat dinner with when you are at home?				
17.	Do you and your family have supper together?				
18.	Are you allowed to eat as much as your friends?				
19.	What do you like to do on your spare time, recesses at school, with your family? On weekends?				

Thank you!
